# Examining the Emotional and Physical Health Impact in Users of Open-Source Automated Insulin Delivery and Sources of Support: Qualitative Analysis of Patient Narratives

**DOI:** 10.2196/48406

**Published:** 2025-01-06

**Authors:** Bryan Cleal, Yanbing Chen, Mandy Wäldchen, Hanne Ballhausen, Drew Cooper, Shane O'Donnell, Christine Knoll, Niklas Krug, Klemens Raile, Tebbe Ubben, Adrian Tappe, Dana Lewis, Ingrid Willaing, Timothy Skinner, Katarina Braune

**Affiliations:** 1 Diabetes Management Research Steno Diabetes Center Copenhagen Herlev Denmark; 2 Embry-Riddle Aeronautical University Daytona Beach, FL United States; 3 School of Sociology University College Dublin Belfield Ireland; 4 #dedoc° Diabetes Online Community Berlin Germany; 5 Brain Simulation Section Department of Neurology with Experimental Neurology Charité – Universitätsmedizin Berlin, Corporate Member of Freie Universität Berlin and Humboldt Universität zu Berlin Berlin Germany; 6 Charité – Universitätsmedizin Berlin Institute of Medical Informatics Berlin Germany; 7 Charité – Universitätsmedizin Berlin Berlin Institute of Health at Charité Berlin Germany; 8 Department of Pediatrics Vivantes Klinikum Neukölln Berlin Germany; 9 AndroidAPS Hamilton New Zealand; 10 OpenAPS Seattle, WA United States; 11 Department of Public Health Copenhagen University Copenhagen Denmark; 12 Australian Centre for Behavioural Research in Diabetes Melbourne Australia

**Keywords:** automated insulin delivery, diabetes technology, type 1 diabetes, insulin pumps, continuous glucose monitoring, peer support, community support, open source, impact, users, diabetes, emotional health, challenges, support, unmet needs, mobile phone

## Abstract

**Background:**

Although commercially developed automated insulin delivery (AID) systems have recently been approved and become available in a limited number of countries, they are not universally available, accessible, or affordable. Therefore, open-source AID systems, cocreated by an online community of people with diabetes and their families behind the hashtag #WeAreNotWaiting, have become increasingly popular.

**Objective:**

This study focused on examining the lived experiences, physical and emotional health implications of people with diabetes following the initiation of open-source AID systems, their perceived challenges, and their sources of support, which have not been explored in the existing literature.

**Methods:**

We collected data from 383 participants across 29 countries through 2 sets of open-ended questions in a web-based survey on their experience of building and using open-source AID systems. Narratives were thematically analyzed, and a coding framework was identified through iterative alignment.

**Results:**

Participants consistently reported improvements in glycemia, physical health, sleep quality, emotional impact on everyday life, and quality of life. Knowledge of open-source AID systems was largely obtained through the #WeAreNotWaiting community, which was also the primary source of practical and emotional support. The acquisition of the components to build an open-source AID system and the technical setup were sometimes problematic.

**Conclusions:**

The #WeAreNotWaiting movement represents a primary example of how informed and connected patients proactively address their unmet needs, provide peer support to each other, and obtain results through impactful, user-driven solutions. Alongside providing evidence on the safety and efficacy of open-source AID systems, this qualitative analysis helps in understanding how patients’ experiences and benefits range from psychosocial improvements to a reduction in the burden of managing diabetes.

**International Registered Report Identifier (IRRID):**

RR2-10.2196/15368

## Introduction

### Overview

In recent years, much attention has been given to the beneficial impacts that online peer support has on people living with chronic health conditions, yet the exact nature of these impacts may still appear to be somewhat intangible [1]. Through our findings, we provide an example of peer support in which the focus of the interaction is very tangible and where the impacts are profound and wide-ranging. The case in point being type 1 diabetes (T1D), where, in recent years, patients have been taking on the role of innovators in the design and development of technology used for their treatment. People with T1D who have developed and disseminated open-source automated insulin delivery (AID) systems exemplify a potential within online peer-to-peer communities that is only just beginning to be realized, particularly among people with chronic health conditions. To better understand how developments have reached the cutting edge in T1D treatment, we first provide some background about the challenges of everyday diabetes management and how open-source AID technology holds promise to alleviate them.

### Background and Challenges in T1D Management

T1D is a lifelong condition caused by the autoimmune-induced loss of insulin-producing cells in the pancreas. Until the discovery of insulin by Banting et al [2] a century ago, T1D inevitably resulted in death by ketoacidosis within months. This changed once pharmaceutically procured insulin was available. Yet, while developments of the last 100 years in pharmaceuticals and technology have improved the physical health and life expectancy of people with T1D from a biomedical perspective, the burden of managing the condition remains a challenge. Life with T1D exists in the center of a continuous data feedback loop, where dosing of exogenous insulin via subcutaneous injections or insulin pumps must be frequently adjusted in accord with glucose levels and predicted trends, carbohydrate intake, physical activity, individual physiology, and behavior, among a variety of other factors.

### The #WeAreNotWaiting Movement

Given the complexity of diabetes management, the everyday experience of managing the condition may lead to frustration, a feeling that was also a key driver in the emergence of the movement that has subsequently become known as #WeAreNotWaiting [3]. Initially, this frustration was related to issues with the accessibility of data from continuous glucose monitors (CGMs) in real time. Taking matters into their own hands, individuals reverse engineered commercially available devices to enable uploading of device data to the cloud in real time. In a model of diffusion that has characterized subsequent developments, the source code was published as open-source software, freely available to anyone and linked by the social media hashtag #WeAreNotWaiting. Eventually, a large, global community has united under this banner.

One of the most significant innovations to emerge from the #WeAreNotWaiting movement are open-source AID systems—sometimes referred to as do-it-yourself artificial pancreas systems—such as OpenAPS [4], AndroidAPS [5], iAPS [6], and Loop [7]. These systems link CGM sensors and insulin pumps with predictive control algorithms running on smartphones or microcontrollers. Insulin dosing is automatically adjusted according to predictions based on real-time data from CGMs, individual settings, and user inputs such as meal information. This technology emerged well in advance of the recent availability of commercial AID systems in select countries [8].

### Current Evidence and User Perspectives

Evidence from randomized controlled trials and observational studies has demonstrated the safety and efficacy of commercial AID systems [9], as well as a positive impact on the users’ lived experience and quality of life [10]. Given the lengthy, complex development and approval processes, only few systems are currently licensed, and their functionality is limited due to regulatory constraints. Even in countries with market availability of commercial AID systems, they are not universally accessible to the user, with reimbursement policies for devices varying considerably between countries [11].

Open-source AID systems work much like commercial AID systems at a basic level, connecting devices and automatically adjusting insulin dosing via predictive algorithms. They differ from commercial systems in terms of device choice, interoperability, transparency, access, customization, and usability. While commercial systems traditionally include manufacturer-designed education for clinicians and users, open-source AID user support and education initially took place via peer support outside of clinical settings [12]. The source code for open-source AID systems is freely available from online portals. In addition, these communities have also created documentation that has been translated into many different languages. Peer support is freely available to help with the setup and use of these systems.

It is estimated that >10,000 individuals worldwide are currently using open-source AID systems, and the uptake continues to increase globally. Evidence from a recent randomized controlled trial [13] and real-world studies based on self- and caregiver-reported outcomes [14,15], in silico [16], user-provided data [17], and observational studies [18-20] point to the safety and effectiveness of these systems, with improvements in clinical parameters such as glycated hemoglobin (HbA_1c_), time in range, occurrence of hypoglycemia, and glycemic variability. However, until recently, the perspective of the user and their reported outcomes has only been touched upon [21-25], and there remains much to learn about how open-source AID systems impact the lives of those who use them.

### Study Objectives

The focus of this study was to establish the physiological, cognitive, and emotional impact of open-source AID system use. We further aimed to identify sources of support and challenges associated with setup, regular use, and maintenance of open-source AID systems. This study was designed and conducted by the OPEN (Outcomes of Patient’s Evidence with Novel, Do-it-Yourself Artificial Pancreas Technology) consortium [26], an international, interdisciplinary team of patient innovators, clinicians, and scientists, many of whom also live with T1D and use open-source AID systems.

## Methods

The research data were obtained from responses to open-ended questions included in a cross-sectional, web-based survey examining the use of open-source AID systems. The survey, titled DIWHY, was conducted between November 2018 and March 2019 [26].

### Survey Design

The survey (Multimedia Appendix 1) was created by the OPEN consortium in collaboration with further open-source AID system users and was piloted with a small group of them before final release. The Checklist for Reporting Results of Internet E-Surveys was used to guide survey development [27]. The survey comprised 39 items in total with 2 sets of open-ended questions, which sought to capture lived experiences with open-source AID systems in the form of narratives. For these questions, participants could provide a free-text response with a maximum length of 1000 words each. The first set of questions assessed sources of information, support, and emotions associated with the preparation and setup process. The second question set addressed the impact of open-source AID system use on everyday life, associated changes, and challenges with respect to the transition to open-source AID systems.

### Participants and Recruitment

Participants included in this study were adults (aged >18 y) who are living with diabetes (type 1, type 2, or other) and are current users of open-source AID systems. There were no restrictions in time since diagnosis or commencement of open-source AID systems. In this study, participants were exclusively recruited from the global diabetes online community, leveraging the outreach of the #WeAreNotWaiting movement. This approach enabled us to tap into a highly empowered and informed population of people with diabetes and particularly target those who were users of open-source AID systems. We used several social media channels, including the Facebook (Meta Platforms) groups Looped (with >6000 members) and AndroidAPS users (with >1800 members as of November 2018) and regional subgroups, and posted publicly on Twitter using the hashtags #WeAreNotWaiting and #DIYAPS to engage with the wider diabetes online community. Participation was further promoted through announcements on the OPEN project website. There were no paid promotions or targeted advertising on any platform. Participants were able to choose between 2 language options (English and German).

### Data Collection and Analysis

Data were collected and managed using REDCap (Research Electronic Data Capture; Vanderbilt University) [28], and the qualitative analysis of the narratives was performed using NVivo (version 12; Lumivero, 2018). The narratives were analyzed with an approach applying the principles of template analysis [29], in which a hierarchical coding structure is recommended to allow researchers to capture the diversity of meaning within broader overarching themes. This approach was deemed necessary due to the framing of the open-ended questions, in which respondents were prompted to reflect on specific aspects of their open-source AID system use experience. Therefore, the initial template and coding were deductively driven, with physical impact, emotional impact, sources of support, and challenges established as the overarching themes. In the subsequent stage of coding, the deductively coded data were analyzed inductively to identify subthemes within the overarching framework. Initially, 3 coders worked (BC, YC, and MW) on a small sample of the deductive data and presented putative inductive themes for evaluation within the author group. These were discussed and refined to ensure that there was equivalence in relation to the levels of abstraction and thus in the hierarchical organization of the template. On the basis of this initial inductive coding, it was agreed that only 1 level of abstraction was necessary and that the overarching themes could be articulated sufficiently by 1 level of subthemes. The resulting comprehensive codebook, which included both deductively and inductively developed codes, descriptions, example quotes, and thematic categories, are detailed in Tables 1-4. All the data were then coded deductively in accordance with the full template by BC and YC, although emergent themes not established in the initial inductive analysis were also proposed. Finally, 2 coders (HB and DC) reviewed the coding template to assess the external validity of the coding process. Where clarity or consistency was questioned, further group discussions were held until all issues were resolved.

**Table 1:** Comprehensive codebook detailing thematic analysis of open-source automated insulin delivery system user narratives collected from a multinational cohort of 383 participants. This table presents findings related to the physical health benefits and quality of life impact of using open-source automated insulin delivery systems, such as improvements in glycemic control, time in range, and reduced hypoglycemia.

Theme		Description	Example quotes
**(A) Physical impact and quality of life impact**			
	Glycemic outcome improvement (A1)	Refers to improved time in range and HbA_1c_^a^ levels, less glucose variability	“I purchased the Miaomiao brand Bluetooth transmitter to send my blood glucose levels to my phone, thereby having access to alerts that have undoubtedly saved my life.” “My biggest hope was to control my blood sugar peaks due to gastroparesis and this works 95% of the of time very well. Thanks to this technology, I am a big step closer to my goal.” “He’s not T1, but was concerned about my time in range and felt the DIY Loop system was better than approved FDA methods.” “Looping has dramatically improved time in range and how I feel. After 5 weeks Looping, my A_1C_ lowered from 6.8 to 6.3.”
	Hypo- and hyperglycemia (A2)	Refers to fewer hypo- and hyperglycemia and reduced complications associated	“Overall, my blood sugar adjustment has improved tremendously. Hypoglycaemia is extremely rare. Times above the limit occur but are limited in duration.” “My values have become immensely better. Hypos with unconsciousness did not occur anymore.” “I’ve always had problems with hypos. The Freestyle showed me in the morning that I was hypoglycemic, nevertheless, I spent the night—retrospectively—in hypoglycemia. Or even a whole night with levels over 250 mg/dl.”
	Sleep quality (A3)	Denotes all aspects of improved sleep quality for either caregivers or children, such as increased sleep duration, fewer sleep interruptions, and feeling better rested in the morning	“Thereby having access to alerts that have undoubtedly saved my life, both while sleeping and while alone in public transportation, among others.” “Only looping for about 2-3 weeks. So far, better quality sleep for my husband and me (no nocturnal hypos which would happen regularly prelooping).” “I sleep so much better. I no longer fear lows. I understand my body’s needs much better.” “It was hard to convince my wife that I was going to turn over control of my diabetes to open source code that I downloaded from the internet. It actually got to a point where I explained to her that I was doing it with or without her approval. When she realized how passionate I was about it, we sat down together and I explained the ins and outs of everything. She sleeps so much better now knowing I’m healthier overall and more safe overnight.”
	Exercise management (A4)	Refers to the improvements in physical exercise since the use of DIYAPS^b^	“Better sleep and exercise management, AAPS has very good objectives to work through. Diabetes management is now less hassle and lower risk.” “Exercising and working out is a lot more easier since I no longer have that many low BGs. I overall feel physically and mentally much better since my time in range has increased by 30%.”
	Quality of life (A5)	Refers to the mentioned improvements in quality of life and describes the degree to which an individual is healthy, comfortable, and able to participate in or enjoy life events	“But today, at 44, I have a system that already reduces a lot, and ensures very good values and thus a better quality of life.” “Help with stress situations both high and low, preworkout and postworkouts are less drastic, overall better mental health since I don’t need to make so many decisions throughout the day about BGs and knowing that I have the ability to have more time in range and a better A_1C_ while still living a pretty normal life.” “A DIY system gives me this flexibility and adaptability. A huge weight has been lifted off my shoulders knowing that I will be safe during the night, and won’t be doing undue damage to my organs during stressful situations like exams or during panic episodes.” “Since then, every night was like that and every day I feel more active and fitter...that’s what I call Quality of Life.”

^a^HbA_1c_: glycated hemoglobin.

^b^DIYAPS: do-it-yourself artificial pancreas systems.

**Table 2:** Comprehensive codebook detailing thematic analysis of open-source automated insulin delivery (AID) system user narratives collected from a multinational cohort of 383 participants. This table summarizes user-reported experiences regarding the positive and negative emotional effects of open-source AID systems.

Theme				Description		Example quotes
**(B) Emotional impact**						
	**Positive (B1): describes positive emotions of participants related to the experience with open-source AID in daily use, including anticipation, enjoyment, excitement, relief, freedom, and inspiration.**					
		Anticipation	Describes hopeful emotional states of anticipation and great expectations of participants that lie on the open-source AID system for improved diabetes management and hope for improved quality of life		“Looking forward to next steps: predicting algorithms.” “I had high hopes that everything would be different from now and I do not have to do anything for my diabetes anymore, and that the loop regulates everything on its own.” “Hope and impatience since I was looking for an atomization almost for 20 years.”	
		Enjoyment	Describes that participants enjoy using the open-source AID system or enjoy the satisfied outcomes brought by open-source AID system use		“Now I enjoy quiet nights and hope for a future without long term complications.” “I am doing well and I am happy to use the loop.” “My CGM alarms are all turned off now and I love the silence.” “In terms of expectations, I had a vague feeling that closed looping could be a solution to my lack of control during the night but I did not have very clear or measurable expectations. I just wanted to try it, I was curious to see what it was going to bring me. And now, of course, sooooooo glad that I did that!” “Just awesome. By far the biggest impact has been the reduction in time spent “doing” diabetes. I am rarely woken at night even after intense exercise and little tweaks which previously interrupted my day have largely disappeared. It is a revelation.”	
		Excitement	Describes exciting emotions of participants related to the experience with open-source AID systems in daily use, including excitement, happiness, and satisfaction with AID system use and associated results		“I was just so excited to learn about DIY closed looping, and ordered gear immediately.” “I have been looping for only 4 months now and I am really grateful to all the people that have worked so hard to developed this amazing technology.” “I was very excited learning about how the system worked. I saw it as a challenge to understand and build it. Understanding the algorithm and building it myself gave me a great understanding of the system and mitigated any fears that I might have had about it’s functioning.” “To me, loop is a miracle—I can hardly believe it after 35 years to finally have normal blood glucose levels.” “Just awesome. By far the biggest impact has been the reduction in time spent “doing” diabetes. I am rarely woken at night even after intense exercise and little tweaks which previously interrupted my day have largely disappeared. It is a revelation!” “[I am] able to read the blood sugar over a watch and no longer have to perform finger pricks, should become true! I still remember the exact moment, I immediately had tears in my eyes.”	
		Relief	Describes that participants feel relieved since their diabetes-related complications alleviated and quality of life improved after using open-source AID systems		“I felt well and relieved, within a few days there was a clear improvement in my control.” “It’s the reduction in disease burden, reduction in lows and highs and the overall improvement in time in range that has changed things so much for me.” “Since the change, I feel safer and simply more comfortable.” “My diabetes does not bother me any more, I can accept it and even like it.”	
		Freedom	Describes the feeling of freedom since participants gained more control of their diabetes by using open-source AID systems		“The essential feeling of freedom and the feeling of being in control of diabetes.” “I feel free for the first time in years!”	
		Inspiration	Describes that participants were encouraged and motivated during their journey of building open-source AID systems		“No special ‘key events’ other than reading many stories of a diverse group of T1’s who all seemed to overcome all the I-am-new-to-software-building challenges and reading how happy they now we’re that they took this effort. Also, reading questions of people and seeing that they were answered fast and elaborately by more experienced and knowledgeable users made a great impression on me.” “I was immediately thrilled and the hints that the whole thing wouldn’t be completely legal somehow motivated me even more.” “I knew that other people could do it so I was convinced I should be able to do it myself as well. I expected to run into issues in the building process, but I wasn’t held back as I knew I would also run into solutions.”	
	**Negative (B2): describes negative emotions, such as fear, frustration, worry and anxiety of users, mainly due to the concern of not being able to build and maintain open-source AID systems and difficulties encountered.**					
		Caution	Describes participants’ concerns in regard to building and maintaining open-source AID systems		“I was quite worried about trying it, there were many things I didn’t quite understand and found technically challenging” “I am still a bit afraid to close the loop.” “I have other fears: will my OTG cable have a loose connection again tonight? will I get to an old replacement pump (combo)? We integrate the Dexcom G6 with xdrip or disassembled xdrip the battery - how was it read today? Will the pharmaceutical industry put more obstacles in our way?” “I have had some fears of system failure, but i do not have them anymore”	
		Frustration	Describes participants’ frustration when experiencing difficulties in sourcing, building, or maintaining open-source AID systems		“Unfortunately, I first failed because of the technology (availability of the accessories, order from the USA). It was a bit frustrating, but I did not want to give up.” “I also experienced a sadness for how long I had struggled (43years) with inadequate tools to manage my condition.” “My biggest challenge has been the isolation of it all. Online support is always present very timely, but not the same as having someone to be present to help troubleshoot when loop stops. I live in very rural area and sometimes I feel like I’m stranded on an island. Frustrating at times, but I would not trade my cure for anything!!”	
		Worry and anxiety	Describes that participants worry about the problems that may arise from the use of open-source AID systems		“as I have no idea what the programme is doing and every time I keep my fingers crossed that I won’t see any screen other than in the tutorial because I have no idea how to fix that.” “The biggest hurdle was I was not confident I could build the loop app on my own.” “And I have concerns about the dependence on Bluetooth/Wifi/Internet/ servers.” “I had heard things about DIY closed loop systems in the various cgm related Facebook groups, but had always thought I wouldn't be able to build & maintain one.” “It was something that I considered at the time, but never thought I would be able to do it as I'm not very good with coding/tech.” “suddenly relying on the phone instead of your own mind is a little strange, I often did not trust the loop and delivered boluses on my own or ignored the TBR suggestions.” “A defective libre sensor the other day (already on the 2nd or 3rd day) has given me a lot of restlessness and effort, because the measurements were incorrect every now and then. I was Worried every now and then a single component of the overall system will fail and cause high adjustment effort.”	

**Table 3:** Comprehensive codebook detailing thematic analysis of open-source automated insulin delivery (AID) system user narratives collected from a multinational cohort of 383 participants. This table outlines the challenges users face during setup, regular use, and maintenance of open-source AID systems, including technical difficulties and knowledge gaps.

Theme			Description	Example quotes
**(C) Challenges**				
	**Accessibility (C1): describes the challenges encountered by the participants regarding accessing the open-source AID systems device, including sourcing hardware, cost, and understanding of the open-source AID systems rationale.**			
		Sourcing hardware	Describes the problem of sourcing hardware that is compatible with open-source AID systems	“My main struggle was with losing access to my favorite pump (OmniPod). I switched back to a tube based pump I'm favor of this system, while hoping that the pods are cracked soon. I followed the OpenOmni efforts.” “but then I was unlikely to do it as I didn’t have the right pump anymore” “And my health insurance was vague about the info they needed to get the pump and supplies reimbursed. Denied my request several times. Took a lot of communication between health care providers, myself, insurance which was a frustrating process for me.” “My health care providers initially didn’t want to prescribe the (loopable) pump.”
		Cost	Describes the issue of costs associated with building open-source AID systems	“The main challenge has been self-funding the CGM which is expensive in New Zealand.” “I started with the pump therapy just before loopin. The health insurance did not supply me with a pump at the time, so I was on my own without diabetic care.” “The Dana RS pump has the one huge advantage of the open interface to control, otherwise the pump looks like a product from the penultimate decade, compared to other systems. The needles are not great either and the counterclockwise luer lock should be replaced. The should be a standard for pump connections to avoid manufacturer tie-ins and make cost reductions possible (the equipment is still too expensive).”
		Understanding	Describes problems with understanding the rationale provided by the instructions for open-source AID system setup	“My initial feeling of starting to build my loop was that it is poorly documented and difficult to diagnose setup issues.” “Understanding the system was both challenging and frustrating. The documentation is poorly explained and the system has its limitations but it is the best we have.” “In the meantime there is a ton of information about closed loops, which was an intensive learning and reading phase in both the medical literature and the posts and documentation of the community. Since at the beginning the overview was missing, this was also a bit confusing because the meaning of individual components was not yet estimable for the own implementation when reading. What is a Wixel, can it be eaten, and if so, how many carbs do I have to expect?. It had to burst a lot of knots until it was clear which software components to make the (actually quite simple) beginning. But maybe that's just too complicated for me, a certain tendency is not to be denied.” “In the lead up to building, I felt overwhelmed by all info.” “Apart from the setup difficulties and my lack of English skills I had no difficulties.”
	**Setting up (C2): describes the difficulties encountered by the participants when maintaining the use of open-source AID** **systems, including adjusting and fine-tuning, consumption of time and effort, inconvenience in everyday life and technical issues.**			
		Adjusting and fine-tuning	Describes the process of determining the factors and further adjusting and fine-tuning	“Determining the factors with decimal place accuracy, calculating the sensitivity factor was/is in first place effortful.” “The only difficulty in the change of therapy (apart from the unfamiliar handling of the technique in general) was to adjust the loop for different types of sports, depending on the time of day and physical condition.” “disillusionment followed immediately - even the updated nightscout / set up is very complicated to operate for non-nerds. It’s often not clear what happens and what is shown on night scout.”
		Time and effort	Describes the amount of time and effort required for open-source AID system setup	“It takes a lot of time and attention to begin with” “It has been a tricky process, as we have been learning by doing” “With the technical implementation, it was an uphill battle. It took a while to work but was worth the effort...I do not want to be without loop...”
		Everyday life	Describes inconvenience with using open-source AID systems in daily life regarding exercise and diet, etc	“I had to reduce the overall carb intake in order to achieve better time in Range, and this had a major impact on my and my family lifestyle” “The regulation of sports is still a bit difficult” “I discovered Loop app was terrific, but the phone was too bulky. So I purchased a new pump outright and started AAPS with a tiny phone. I am actually about to try AAPS on a stand-alone watch. The bulk of the gear is super important to me, as I can't be carrying a lot of excess stuff at work” “I always think about if I need to charge anything (with Enlite it’s just a bit more complicated than with other systems.) I have to keep reminding myself not to go out of the house without my new hand bag - and not without a smartwatch” “Social judgment from part of the DT1 Community which [accused] me of obsession with the disease.”
		Technical challenges	Describes technical issues with the equipment, such as battery charging	“The issues I’ve had have been of a minor technical issue only, like accidentally shorting out my Miao Miao charger, and having difficulty ordering another. So I changed to Dexcom G5, and have learned to rebattery my transmitters, making it actually cheaper than using Libre. I have also had some troubles with my phone updating it’s OS and becoming useless. I also have some battery issues with my new pump.” “Biggest challenge was building the app in Android studio, as I have no idea what the programme is doing and every time I keep my fingers crossed that I won't see any screen other than in the tutorial because I have no idea how to fix that.” “The only difficulties that I am sometimes experiencing are technical problems such as connectivity issues between the Riley link and the pump, and blood sugar fluctuations once the insulin sensitivity changes, which are more noticeable now with a tighter BG management than before, where every day with diabetes was just chaos.”

**Table 4:** Comprehensive codebook detailing thematic analysis of open-source automated insulin delivery system user narratives collected from a multinational cohort of 383 participants. This table highlights the role of peer support, online communities, and other resources in facilitating the adoption and use of open-source AID systems, emphasizing the sense of community and shared expertise.

Theme				Description		Example quotes
**(D) Support**						
	Family and friends (D1)			Describes that the participants received support from their family or friends		“My husband supported and encouraged me, which helped but I’d have gone ahead even without that support.” “Friend who had acquired the components and assisted [me] in building. He was already using [AndroidAPS].”
	Online support (D2)			Describes the types of support that participants seek on the web, such as social media, blogs, forums, and other people with diabetes		“First, I found information about AAPS in Freestyle Libre Forum.” “I felt the whole process was very simple with very comprehensive instructions and support through Facebook.” “March 2017 was the first time I started looking for treatment improvements. I acquired all the information and knowledge in through own research on the internet.” “In our area there is a Whatsapp diabetes group that meets in person now and then. A PwD there works in IT and was contacted about 1,5 years ago. With his technical support I have closed my loop.” “I set up a Nightscout server and the AndroidSeries600Upload app for his Medtronic pump in the hospital - and read a lot - and at first set up OpenAPS for myself. As a technophile I was of course immediately on fire, but I still took 2 months to read about the topic online intensively, trawling through forums, Facebook etc., and my anticipation and enthusiasm grew steadily.”
	DIY^a^ community (D3)			Describes that the participants received support from the DIY community, such as help from author DL		“I got support from the DIY community via face to face meetings and via the online community.” “When I could connect the technical side (IT) with my diabetes, it all started. Then I met Adrian and Dana [authors AT and DL] in person.”
	Conference and meeting (D4)			Describes the support that the participants received from conferences or meetings		“However, due to the great help from the looper.de group and the looper meeting, I already wanted much more at this time and have been able to implement this with a lot of reading and informing and with still some technical problems”
	Medical professionals (D5)			Describes that the participants received support from medical professionals, such as doctors, nurses, diabetes educators, endocrinologists, etc		“I actually first heard about DIY options from my doctor, who referred me to another patient who was already using one.”
	Self-support (D6)			Describes that the participants learn to build DIYAPS^b^ all by themselves, with no direct support from others		“I like the user manual, which it written clearly step by step.” “[N]o direct support or advice from third parties.”

^a^DIY: do-it-yourself.

^b^DIYAPS: do-it-yourself artificial pancreas systems.

### Ethical Considerations

This study was performed in line with the principles of the Declaration of Helsinki. Approval was granted by the Ethics Committee of Charité–Universitätsmedizin Berlin (EA2/140/18). Informed consent was obtained electronically from all individual participants included in the study. The deidentified datasets are available from the corresponding author upon request. Participation was anonymous and voluntary. No financial or other compensation was provided.

## Results

### Participant Characteristics

In total, 383 participants (N=722, 53% of participants of the DIWHY survey) responded to the open-ended questions in the survey, and there were a combined 645 responses to the 2 open-ended items. Characteristics and clinical features of the cohort are shown in Table 5.

**Table 5:** Demographic and self-reported health characteristics of the participants using open-source automated insulin delivery (AID) systems.

Demographics		Values
**Gender (n=383), n (%)**		
	Men	203 (53)
	Women	179 (46.7)
	Other	1 (0.3)
Age (y), mean (SD)		43 (12)
**Type of diabetes (n=383), n (%)**		
	Type 1	381 (99.5)
	Type 2	0 (0)
	Other	2 (0.5)
Duration of diabetes (y), mean (SD)		30 (12)
Duration of open-source AID system use (y), mean (SD)		4 (2)
Most recent self-reported HbA_1c_^a^ level, mean (SD)		5.89 (0.62)
**Type of open-source AID system used regularly (n=423), n (%)**		
	OpenAPS	65 (15.4)
	AndroidAPS	245 (57.9)
	Loop	110 (26)
	Other^b^	3 (0.7)
**Region (n=383), n (%)**		
	Europe	282 (73.6)
	Germany	184 (48)
	United Kingdom	41 (10.7)
	Austria	7 (1.8)
	Spain	7 (1.8)
	Netherlands	4 (1)
	Finland	6 (1.6)
	Czech Republic	5 (1.3)
	Bulgaria	4 (1)
	Sweden	2 (0.5)
	Other^c^	22 (5.7)
	North America	69 (18)
	Canada	15 (3.9)
	United States	54 (14.1)
	Western Pacific	23 (6)
	Australia	12 (3.1)
	New Zealand	11 (2.9)
	Asia	2 (0.5)
	South Korea	2 (0.5)
	Africa	2 (0.5)
	Algeria	1 (0.3)
	South Africa	1 (0.3)
	I’d rather not say	5 (1.3)
**Education (n=379), n (%)**		
	Doctorate or graduate degree	154 (40.6)
	Bachelors, professional or associate degree	129 (34)
	Trade, technical or vocational training	27 (7.1)
	Some college credits	11 (2.9)
	Secondary school	34 (9)
	Some secondary or primary school	22 (5.8)
	No schooling completed or none of the above	2 (0.5)
**Occupational status (n=382), n (%)**		
	Full time	265 (69.4)
	Part time	60 (15.7)
	Unemployed	4 (1.1)
	Retired	20 (5.2)
	Student	24 (6.3)
	Other or none of the above	9 (2.4)
**Professional background (n=305), n (%)**		
	Medicine	76 (24.9)
	Tech	81 (26.6)
	Finance	40 (13.1)
	Other	108 (35.4)
**Household annual income (US $; n=336), n (%)**		
	<20,000	34 (10.1)
	24,000-34,999	27 (8)
	35,000-49,999	48 (14.3)
	50,000-74,999	87 (25.9)
	75,000-99,999	44 (13.1)
	>100,000	79 (23.5)
	I’d rather not say	17 (5.1)

^a^HbA_1c_: glycated hemoglobin.

^b^xDrip, Nightscout, offline uploader for Medtronic 600 series, Hackabetes Artificial Pancreas Project, custom, or own developments.

^c^Belgium, Croatia, France, Hungary, Ireland, Italy, Lithuania, Poland, Romania, Russia, Serbia, Slovakia, and Switzerland.

### Template Analysis

#### Emotional and Quality of Life Impact

“Anticipation” and “curiosity” were emotions mentioned by participants in relation to their first encounter with open-source AID technology. This highlights the intuitive appeal of this solution for diabetes management—“I had envisioned this type of solution for many years and was looking out for the emergence of suitable technology” (Man, age 59 years, United Kingdom)—and why, for many people, initial reservations were quelled by the potential improvements offered by AID systems—“One is a little uncertain, but the curiosity for the improvement of control has won!” (Gender unspecified, age 49 years, Germany).

However, as this sentiment indicates, anxiety and caution were also a part of the emotional responses experienced by our participants. This could be as they confronted the prospect of developing their own system—“I was quite worried about trying it, there were many things I didn’t quite understand and found technically challenging” (Woman, age 69 years, United Kingdom)—but was also apparent even once the system had been successfully built—“Originally it felt like a big step to let the algorithm make changes” (Woman, age 42 years, Australia).

Thus, both the challenges—real and anticipated—in setting up the system and the prospect of allowing an algorithm to undertake a life-critical role could have a negative emotional impact.

However, for the most part, initial concerns about the complexity of the technology and ceding control to an algorithm were replaced by a sense of “pride” and “relief”—“I feel very good and proud I did it because it was technically difficult to build it with my pump and CGM” (Man, age 50 years, Germany).

The sense of relief experienced by our participants was 2-fold; relief that the system was built and functioning but also a sense of being partially released from the burden of everyday diabetes management—“The most impressive thing is how little diabetes suddenly plays a role, how simple everything has become, how rarely one suddenly has to wonder about metabolic fluctuations, how well one can sleep, knowing that blood sugar stays in range” (Woman, age 49 years, Germany).

Twisting the concern with automation, some participants also noted that it was precisely because control was given over to an algorithm that improved outcomes could be achieved—“I was happy to hand over control to something which makes fewer irrational decisions and is less emotionally involved in the process” (Woman, age 35 years, United Kingdom).

The relief they experienced did not come without considerable effort and “frustration,” and this was also a common emotion in the narratives. Part of this frustration was related to the reliability of technology:

While the burden of what to do in reaction to blood glucose has gone down, the tech troubleshooting and figuring out how to fine-tune has increased greatly. Traded one problem for another.

Woman, age 62 years, United States

As can be inferred from this comment, frustration was also driven by an expectation that the level of automation would be greater: “A few months into closed looping I am starting to see results, though I was expecting [it] to be easier and thought it was going to handle much more the ups and downs by itself” (Man, age 41 years, Netherlands)*.*

Yet, most participants declared themselves happy to invest the effort when the reward was so tangible and transformative for overall quality of life:

...it doesn’t just fix everything and that there are still settings to adjust and check, but once these were okay then I’ve had very few issues. It has allowed me to take a back seat with my diabetes care...It has taken huge amounts of the diabetes burden away from me!

Woman, age 25 years, United Kingdom

In fact, the work also served as a source of inspiration, with many participants gaining new insights into important factors influencing glucose fluctuations:

Looping has provided me much detailed insight into the inner-workings of my endocrine system and diabetes management. I’ve learned that my insulin ratios and [basal rates] needed to be greatly adjusted. As I’ve learned, two bad settings can mask each other and end up appearing to be correct. The learning curve is steep, but very rewarding.

Woman, age 24 years, France

Overall, participants indicated that the net gains of open-source AID were extensive and profound. Often, a sense of gratitude was expressed:

The community has helped me so much. I can’t express my gratitude to all developers, helpers and people in my local community as well who freely give their time and skills to make this possible.

Woman, age 65 years, Australia

#### Source of Support

The community mentioned earlier highlights the particular model of diffusion that has fostered the use of open-source AID systems. To echo a common refrain in this context, *do-it-yourself* does not mean *do-it-alone* [15], so while each user is ultimately responsible for building their own system, the support that they can obtain in doing so is potentially extensive.

For those without preexisting skills in IT, support was at hand, for some, among one’s established social network, for example, family and friends:

I was very intimidated at first as I have extremely limited coding knowledge. After following along in the group for a while, I began to get more comfortable. My boyfriend also encouraged me and offered to help set it up since he has a bit more tech knowledge.

Woman, age 22 years, Canada

Both technical and medical expertise within the individual’s network was an important antecedent to the uptake of open-source AID systems:

My partner immediately supported me because, as a doctor, it was very clear to him that it had a much better metabolic effect...The support of the Facebook group especially for small logistic things was very important to me.

Woman, age 37 years, Germany

This participant’s comments also highlighted that direct support from health care professionals (HCPs) may be lacking:

The support by my social environment has increased, the support by doctors and the diabetologist’s office are completely lost, I consider this to be a risk, I am waiting for the moment when our diabetologists will not only be “not forbidden” but required to inform about the Closed Loop as the gold standard of therapy.

Woman, age 37 years, Germany

This is not to say that HCPs were not supportive or positive about AID outcomes, but the support they could provide was more often informal and emotional rather than practical:

My two diabetologists know about the loop and are amazed/enthusiastic about my [glucose] levels, but unfortunately cannot support me for legal reasons.

Woman, age 55 years, Germany

While an individual’s social relations could profoundly impact the building and maintaining an open-source AID system, support was still available even without direct expertise in one’s personal network—“I’ve found the technology almost impossibly difficult to deal with and have had a considerable amount of personal help from other users” (Woman, age 62 years, United Kingdom).

For some users, this occurred at face-to-face meetings (eg, “build events”), where expert users could guide them, but, for the majority, such support was obtained via online forums—“The biggest (and for me only) help with technical problems or ‘fine-tuning’ the settings is provided by the Looper online community” (Woman, age 27 years, Germany).

The #WeAreNotWaiting community was the main source of support cited by our participants, and this was multifaceted. In its most basic form, the online documentation developed by users for users was an essential resource and frequently praised for its clarity. Beyond this, in the various social media–based groups connected with open-source AID systems, there was also a wealth of information from reading existing threads and others’ posts, where frequently asked questions and troubleshooting topics could guide through challenging aspects of the process. Finally, the online forums also served as a real-time support network, where users could expect rapid and reliable responses to whatever issue they might reach out for:

I don’t want to finish without mentioning the importance that the support groups are having to me. Both in helping understand and setting the system and managing the everyday life... It is completely amazing being able to be connected to so many people who are also looping and give and get support.

Woman, age 40 years, Spain

Also notable was how the encounter with this community and its essentially altruistic spirit could inspire new users to be willing to participate and serve within this support network:

What one cannot do, the many can manage. The group has helped me. I'm getting involved as well and spread the knowledge so others can benefit from it.

Man, age 54 years, Germany

#### Challenges

Principally, the challenges reported included (1) accessibility and (2) technical setup and maintenance. There were 3 prevailing issues regarding accessibility. The first one was cost, since essential hardware was not readily available via health care services or insurers:

CGM is prohibitively expensive in my country. I only started using it two months before looping as part of preparing to loop. I am trying to hang in there paying for it because of the fantastic benefits but it is a big drain on family income.

Woman, age 51 years, Australia

In addition, even in circumstances where hardware was potentially available to users via public health care or insurance, access could still be problematic if potential users were not eligible according to insurers, or HCPs had reservations about recommending devices that could be used for open-source AID systems:

My healthcare providers initially didn’t want to prescribe the (loopable) pump. And my health insurance was vague about the info they needed to get the pump and supplies reimbursed. Denied my request several times. Took a lot of communication between health care providers, myself, insurance which was a frustrating process for me.

Woman, age 33 years, country of residence undisclosed

The final aspect of accessibility was the anticipated complexity of the process and the documentation to be followed to set up an open-source AID system—“I had heard things about DIY closed-loop systems in the various CGM-related Facebook groups, but had always thought I wouldn’t be able to build & maintain one” [Woman, age 47 years, United Kingdom].

Thus, the obstacle was sometimes more about expectation than experience, with people deterred from the attempt by the expectation that they would not be able to solve technical issues.

However, for others, it was as much the experience as the expectation that could provide an obstacle to access—“There was a lot to learn. I often sat crying in front of the computer” (Woman, age 35 years, Germany).

Technical challenges associated with building open-source AID systems were prevalent in the narratives, with only a handful indicating that the process was straightforward:

I tried about a year before I actually started to build a system and it proved too difficult. After a year of being burned out and things not being any better, I tried again and succeeded.

Woman, age 36 years, United States

Participants conveyed difficulties with both hardware and software components, for example, with connectivity loss.

Even with all components fully functioning and connected, other technical challenges remained, though these were more related to the technicalities of diabetes management than technology per se. Users of open-source AID systems take on the role of diabetes experts as much as programmers and are required to fine-tune the settings on their devices in accordance with a selection of parameters:

[The] first weeks of looping were a bit hard because my ratios were off and it was hard to understand why Loop is making some decisions. Or, what’s even more important, which parameter should be tweaked in order to make it behave better.

Man, age 32 years, Poland

Determination of these settings is generally undertaken by HCPs in the context of prescribed, commercial devices, ideally in collaboration with the user, but it may remain more or less opaque to the individual with diabetes. So, although many users were highly engaged with diabetes management previously, there was a learning curve involved for most, not least because the level of control for different parameters allowed by open-source AID systems extends significantly beyond those in commercial systems:

So I have to work more on my settings. Nothing is (fully) automatic and runs all by itself. For me, as a technician who believes in the possibilities of self-regulating automation, there is still a lot to be done.

Man, age 51 years, Germany

The combination of the different challenges involved in building an open-source AID system evoked another issue for some, inasmuch that considerable time was required to resolve the issues emerging from building and maintaining the system—“My husband has suggested several times that I was doing more work with the system than without, because of the frustration & time (whole weekends) involved in getting my loop back up & running” [Woman, age 51 years, United States]*.*

Yet, while time was undoubtedly a factor to be dealt with, the extent to which it was perceived as a challenge, impinging on everyday life, was often weighed against the time spent “doing diabetes” before transitioning to open-source AID systems:

My own personal tight control prior to looping was very time-consuming. Post APS I save more than 1hr every day. Imagine my experience of living 1/24th longer life for the rest of my life because of APS.

Man, age 46 years, New Zealand

#### Physical Impact

For the most part, participants reported marked improvements in physical health in accordance with the measures traditionally used to gauge this in T1D, such as HbA_1c_ and time in range, as reported elsewhere [15]. For some, these improvements occurred soon after their switch to open-source AID systems:

Benefits from the first 8 weeks: 80-90% of the time-in-range without changing my lifestyle! Previously that value was 40-45%.

Man, age 37 years, Germany

The time regarding changes in HbA_1c_ was longer but still commented upon, often as levels within a reference range for people without diabetes:

I knew some that tuning was needed but I was patient. Now I have used a DIY system 24/7 approx 23 months for almost two years! Hba_1C_ is 5.2%, I’m happy.

Man, age 44 years, Finland

In addition to these clinical outcome improvements, participants also reported changes in their health based on more immediate, everyday experiences. The experience of hypoglycemia was something alluded to extensively—“The blood glucose fluctuations and the hypos have become much less, I feel much safer and I am doing things again that I used to avoid” (Woman, age 45 years, Germany).

The sense of safety can have a profound effect on an individual’s life. Both hyper- and hypoglycemia in their moderate expression can induce physical symptoms that are unpleasant and disruptive but, in their extreme extent, are potentially fatal. Ameliorating the risk of highs and lows, open-source AID systems served to diminish the unpleasant symptoms and, at the same time, reduce the anxiety attached to what might happen in worst-case scenarios. This, aside from its direct benefits, also had follow-on effects on other health-related aspects. Many participants noted benefits of open-source AID systems related to physical activity—“I’m much less afraid of unplanned physical activity because the loop usually regulates it with a few extra carbs” (Woman, age 55 years, Germany).

Again, the point here is not only that glucose levels are within range during exercise but also that the potential fear around exercising was lessened. Fear of exercise and its unpredictable impact on glucose levels represents a clear obstacle removed by open-source AID systems, with potential general health benefits—“I have recently started exercising again after years of sedentary living” (Woman, age 31 years, Australia).

By far, the most frequently mentioned quality of life improvement among our participants was sleep duration and quality:

I can sleep and have no alarms from the CGM at night. In the morning I wake up with a value that I do not have to correct. This has a positive effect on the blood sugar during the day.

Woman, age 38 years, Germany

Persistently disturbed sleep is by any reckoning something that one would expect to impact health and overall quality of life, but for people living with T1D, it is a given:

I SLEEP. That’s the most brilliant, life-changing thing. I’d been sleep-deprived for so long I didn’t even realize what a difference it would make.

Woman, age 49 years, United States

In a similar vein to exercise, open-source AID systems had a dual impact in relation to sleep. It served to alleviate symptoms of hypo- and hyperglycemia that could disturb sleep directly or trigger alarms on devices waking people up. Simultaneously, it helped reduce the fear of nocturnal hypoglycemia, which could result in sleep difficulties due to anxiety and adverse aversion strategies, such as intentionally aiming for higher glucose levels before sleep:

I have no anxiety about sleeping alone when my wife is working away from home. I actually sleep through the night. Eating out is no longer a major stress since I know that even if I underestimate carbs [it] will fix my errors overnight and I will wake mostly in range.

Man, age 42 years, United Kingdom

As might be expected, improved sleep was also associated with further physical and mental health benefits:

Waking up that first morning in a normal range, and every morning thereafter was amazing. It’s incredible how much more you can get done in a day when you wake up in a normal blood glucose range.

Man, age 39 years, United States

Part of this is obviously about having more energy because of being well-rested. Beyond that, there is the important difference of waking up with glucose in target range and how this resonates through the rest of the day:

The almost fully automatic delivery of needed insulin has made life a lot easier and once the factors are set correctly, it is almost possible to live like a ‘healthy’ person. It’s also much easier to start a new day, starting at a value of 90 mg/dl and not 180-200 as before! Working days are much easier than before!

Man, age 47 years, Germany

Rather than using considerable time, energy, and resources attempting to reestablish balance in one’s glucose levels, open-source AID systems allowed people to concentrate more on the business of the day. So, whereas previously a working day may have felt more like “running the gauntlet,” a different experience and outlook on life could be fostered:

Since I’ve used [open-source AID], I was upgraded on my job, I’m mentally faster and sleep like a baby without alarms. I’ve started several personal projects and [I’m] currently on professional certification. I have plenty of quality time now without hypo or hyper and finally happy.

Woman, age 40 years, Spain

## Discussion

### Principal Findings and Their Implications

The findings we present here concerning the impact of open-source AID systems highlight their immense benefits from the perspective of the user, simultaneously setting the extremely challenging nature of diabetes and the ways it may compromise quality of life into relief. This is the first study to analyze narratives and to examine the emotional and physical health impact of open-source AID systems in adult users. Our findings are in line with our analysis of children and adolescents using open-source AID systems, including their caregivers [30], although there were age-specific findings, for example, navigating diabetes throughout puberty and remote monitoring and control by caregivers that only applied to the child cohort. Our results also align with other, smaller cohort studies examining the user experience with open-source AID systems [22,24,31] with the literature pointing to the importance of setting expectations for both onboarding and the ongoing use of AID systems [32,33]. Studies of users of commercial AID systems found similar results [34,35]. Furthermore, the sense of community and empowerment, often referred to as “paying it forward,” was almost exclusively described in open-source AID system users.

In our approach to analyzing the data, we opted to use 4 categories as the basic framework for our template. This was necessary, first and foremost, because these topics were already framed in the wording of the questions. That said, the findings also underline the somewhat fluid nature of the categories, especially with respect to the physical and emotional impact that open-source AID systems can have. So, while we have sought to tease emotional and physical impacts apart for the purposes of our analysis, our findings ultimately serve to highlight how inexorably bound up they are. This is most intuitively illustrated through the example of sleep, where poor quality sleep inevitably impacts emotional well-being, which may, in turn, also impact glucose levels, both directly and indirectly [36].

### Peer Support as a Driver of Innovation and Empowerment

Although there is not the same level of symbiosis between the categories “challenges” and “support,” the findings did point to a strong link between the two in the sense that many of the challenges associated with initiating and maintaining the use of an open-source AID system were resolved via support from a wider community of users. The sense of community underpinning the development and diffusion of open-source AID systems and peer support as a key resource for practical but also emotional support were predominant topics in other qualitative studies on the lived experience with open-source AID systems [22,31]. For many, the discovery of a peer group that one could identify and engage with was as important and meaningful as building their AID system.

As has previously been noted [37], digital platforms can provide opportunities for peer support and the exchange of experiential knowledge about living with illness. The importance of peer support for people with diabetes in the context of online communities has been clearly highlighted elsewhere [12,22,23,38,39]. Moreover, engagement in these communities has been shown to positively impact HbA_1c_ levels [23,40]; reduce diabetes-related distress [41]; and foster support and connection, advocacy, self-expression, information and education, technical support, and humor as a coping strategy [38,42].

However, it is also evident that the type of peer support upon which the dissemination of open-source AID systems has been based is of a somewhat different order. In part, this reflects something of the nature of T1D, where the prevailing model of care, inasmuch as it requires individuals to be actively engaged in their care, may potentially foster the growth and dissemination of expertise [43]. In this situation, the delineation of expertise into “professional” and “laypeople” seems outmoded and evokes the well-known Shavian aphorism that the former serves the purpose of conspiring against the latter [44].

As AID algorithms are being constantly developed further and new features will be introduced (eg, fully closed-loop systems without bolusing for meals), future research should address the lived experiences of people with diabetes associated with their use in addition to the analysis of clinical outcomes. Their full health impact can only be evaluated if real-world user experiences are included.

Looking back at how innovations in diabetes treatment were perceived by HCPs and the scientific community over the last century, increased autonomy and empowerment of people with diabetes have continuously been regarded with skepticism. Similarly, it was debated if people with diabetes were capable of blood glucose self-monitoring in the 1970s [45], calculating their insulin doses by themselves, or understanding real-time readings of their CGM device [46]—all aspects that are standard of care today. In the IT age, people with diabetes creating their own technological tools might be just another iteration of patient empowerment but accompanied by similar controversy. The “lesson learned” from these controversies should be the urgency to foster collaboration with patients and involve them early as stakeholders—whether in research and development of medical devices or the development of care concepts that will ultimately affect them.

This study is the first large-scale qualitative study assessing the lived experiences of adult open-source AID system users. Moreover, it is a study with a truly multinational scope, and in its stakeholder engagement via the involvement of the #WeAreNotWaiting community, it strives to remain true to the values of the phenomenon it is investigating.

Of course it should be noted that the survey was undertaken in 2019, and thus the participants can be considered early adopters of open-source AID systems. At one level, this means that the size of our sample represents what, at that time, was a significant proportion of all users of open-source AID systems. However, by the same token, given the subsequent dynamic technical development and rapid expansion of open-source AID system users since that time, the experiences captured in our study may not be reflective of later experiences and current use of open-source AID systems. In addition, many position papers and an international consensus paper have been published to provide guidance to HCPs who wish to support people using open-source AID systems, which may have contributed to increased knowledge and a change of attitudes [47,48]. A selection bias may be present with the survey only being available in German and English.

### Conclusions

The efforts of the #WeAreNotWaiting community are changing the landscape of available treatment options and the way we look at the role of patients as initiators rather than as passive recipients of a health care product or service. The online communities that support this movement have not only transformed diabetes care with its technology but have also eased the individual burden for those involved due to the tools and peer support it has fostered. The extensive testimony from users of open-source AID systems acquired in this study provides new insights, highlighting factors inspiring people to adopt such solutions, user experiences in transitioning to open-source AID systems, and the transformative impact of AID systems on the everyday life of people with diabetes. These results may contribute to a better understanding of their unmet needs; the impact of AID systems on physical and emotional health; and some of the current challenges to the uptake of AID technology in terms of access, availability, and usability.

## Appendix

Multimedia Appendix 1 DIWHY Questionnaire for Adults.

## Abbreviations

AID: automated insulin delivery
CGM: continuous glucose monitor
HbA_1C_: glycated hemoglobin
HCP: health care professional
OPEN: Outcomes of Patient’s Evidence with Novel, Do-it-Yourself Artificial Pancreas Technology
REDCap: Research Electronic Data Capture
T1D: type 1 diabetes

## Data Availability

The datasets generated and analyzed during this study are available from the OPEN project on request. Researchers interested in working with the OPEN data are encouraged to submit inquiries for data access to the project coordinator (SO) via email. The authors welcome the opportunity to collaborate and accommodate research needs where possible.
