# Correction: Examining the Emotional and Physical Health Impact in Users of Open-Source Automated Insulin Delivery and Sources of Support: Qualitative Analysis of Patient Narratives

**DOI:** 10.2196/72519

**Published:** 2025-03-24

**Authors:** Bryan Cleal, Yanbing Chen, Mandy Wäldchen, Hanne Ballhausen, Drew Cooper, Shane O'Donnell, Christine Knoll, Niklas Krug, Klemens Raile, Tebbe Ubben, Adrian Tappe, Dana Lewis, Ingrid Willaing, Timothy Skinner, Katarina Braune

**Affiliations:** 1 Diabetes Management Research Steno Diabetes Center Copenhagen Herlev Denmark; 2 Embry-Riddle Aeronautical University Daytona Beach, FL United States; 3 School of Sociology University College Dublin Belfield Ireland; 4 #dedoc° Diabetes Online Community Berlin Germany; 5 Brain Simulation Section Department of Neurology with Experimental Neurology Charité – Universitätsmedizin Berlin, Corporate Member of Freie Universität Berlin and Humboldt Universität zu Berlin Berlin Germany; 6 Charité – Universitätsmedizin Berlin Institute of Medical Informatics Berlin Germany; 7 Charité – Universitätsmedizin Berlin Berlin Institute of Health at Charité Berlin Germany; 8 Department of Pediatrics Vivantes Klinikum Neukölln Berlin Germany; 9 AndroidAPS Hamilton New Zealand; 10 OpenAPS Seattle, WA United States; 11 Department of Public Health Copenhagen University Copenhagen Denmark; 12 Australian Centre for Behavioural Research in Diabetes Melbourne Australia

In “Examining the Emotional and Physical Health Impact in Users of Open-Source Automated Insulin Delivery and Sources of Support: Qualitative Analysis of Patient Narratives” J Med Internet Res 2025;27:e48406) the authors noted a few errors in the affiliations.

Author Drew Cooper's affiliations have been changed from:

School of Sociology, University College Dublin, Belfield, Ireland

Diabetes Management Research, Steno Diabetes Center Copenhagen, Herlev, Denmark

to:

Brain Simulation Section, Department of Neurology with Experimental Neurology, Charité – Universitätsmedizin Berlin, Corporate Member of Freie Universität Berlin and Humboldt Universität zu Berlin, Berlin, Germany

Charité – Universitätsmedizin Berlin, Institute of Medical Informatics, Berlin, Germany

Charité – Universitätsmedizin Berlin, Berlin Institute of Health at Charité, Berlin, Germany

Author Christine Knoll’s affiliations have been changed from:

School of Sociology, University College Dublin, Belfield, Ireland

Charité – Universitätsmedizin Berlin, Berlin Institute of Health at Charité, Berlin, Germany

to:

Charité – Universitätsmedizin Berlin, Institute of Medical Informatics, Berlin, Germany

Author Katarina Braune’s affiliations have been changed from:

Diabetes Management Research, Steno Diabetes Center Copenhagen, Herlev, Denmark

Charité – Universitätsmedizin Berlin, Berlin Institute of Health at Charité, Berlin, Germany

to:

Charité – Universitätsmedizin Berlin, Institute of Medical Informatics, Berlin, Germany

Charité – Universitätsmedizin Berlin, Berlin Institute of Health at Charité, Berlin, Germany

The correction will appear in the online version of the paper on the JMIR Publications website on March 24, 2025, together with the publication of this correction notice. Because this was made after submission to PubMed, PubMed Central, and other full-text repositories, the corrected article has also been resubmitted to those repositories.

