# Supplementary material for: Examining the Emotional and Physical Health Impact in Users of Open-Source Automated Insulin Delivery and Sources of Support: Qualitative Analysis of Patient Narratives
Source: J Med Internet Res. 2025 Jan 6;27:e48406. doi: 10.2196/48406 (PMC11747533; doi:10.2196/48406)
Supplement: Multimedia Appendix 1 [file jmir_v27i1e48406_app1.pdf]

DIWHY English for Adults

Record ID ________________________

Welcome to the DIWHY Survey!

This study aims to examine the motivations of current Do-It-Yourself Artificial Pancreas users and to explore the barriers to, and facilitators of, building and maintaining such systems and how they might differ by socioeconomic status, ethnicity, gender and age.

The first part of this online survey consists of 34 multiple choice questions. Additionally, you are invited to reflect on your motivations behind DIY-looping, to share your individual patient journey, and to describe any changes you have experienced in your day-to-day lives.

As part of the EU-H2020 funded "OPEN"-project (Outcomes of Patients' Evidence with Novel, Do-It-Yourself Artificial Pancreas Technology), this study will provide a better understanding of the unmet needs of people with diabetes and current challenges to uptake, which will, in turn, facilitate dialogue and collaboration to strengthen the involvement of open source approaches in healthcare.

Thank you very much for your participation.

Please specify your gender.

- ◯ Female
- ◯ Male
- ◯ Other

Please enter your age in years. ________________________

Please enter your country of residence.

◯ I'd rather not say
◯ Afghanistan
◯ Albania
◯ Algeria
◯ American Samoa
◯ Andorra
◯ Angola
◯ Anguilla
◯ Antarctica
◯ Antigua and Barbuda
◯ Argentina
◯ Armenia
◯ Aruba
◯ Australia
◯ Austria
◯ Azerbaijan
◯ Bahamas
◯ Bahrain
◯ Bangladesh
◯ Barbados
◯ Belarus
◯ Belgium
◯ Belize
◯ Benin
◯ Bermuda
◯ Bhutan
◯ Bolivia
◯ Bosnia and Herzegovina
◯ Botswana
◯ Bouvet Island
◯ Brazil
◯ British Indian Ocean Territory
◯ Brunei
◯ Bulgaria
◯ Burkina Faso
◯ Burundi
◯ Cambodia
◯ Cameroon
◯ Canada
◯ Cape Verde
◯ Cayman Islands
◯ Central African Republic
◯ Chad
◯ Chile
◯ China
◯ Christmas Island
◯ Cocos (Keeling) Islands
◯ Colombia
◯ Comoros
◯ Congo
◯ Cook Islands
◯ Costa Rica
◯ Côte d'Ivoire
◯ Ivory Coast
◯ Croatia
◯ Cuba
◯ Cyprus
◯ Czech Republic
◯ Denmark
◯ Djibouti
◯ Dominica
◯ Dominican Republic
◯ Ecuador
◯ Egypt
◯ El Salvador
◯ Equatorial Guinea
◯ Eritrea
◯ Estonia
◯ Ethiopia

◯ Falkland Islands (Malvinas)
◯ Faroe Islands
◯ Fiji
◯ Finland
◯ France
◯ French Guiana
◯ French Polynesia
◯ French Southern Territories
◯ Gabon
◯ Gambia
◯ Georgia
◯ Germany
◯ Ghana
◯ Gibraltar
◯ Greece
◯ Greenland
◯ Grenada
◯ Guadeloupe
◯ Guam
◯ Guatemala
◯ Guernsey
◯ Guinea
◯ Guinea-Bissau
◯ Guyana
◯ Haiti
◯ Heard Island and McDonald Islands
◯ Holy See (Vatican City State)
◯ Honduras
◯ Hong Kong
◯ Hungary
◯ Iceland
◯ India
◯ Indonesia
◯ Islamic Republic of
◯ Iraq
◯ Ireland
◯ Isle of Man
◯ Israel
◯ Italy
◯ Jamaica
◯ Japan
◯ Jersey
◯ Jordan
◯ Kazakhstan
◯ Kenya
◯ Kiribati
◯ Korea (North)
◯ Korea (South)
◯ Kuwait
◯ Kyrgyzstan
◯ Lao People's Democratic Republic
◯ Latvia
◯ Lebanon
◯ Lesotho
◯ Liberia
◯ Libya
◯ Liechtenstein
◯ Lithuania
◯ Luxembourg
◯ Macao
◯ Macedonia
◯ Madagascar
◯ Malawi
◯ Malaysia
◯ Maldives
◯ Mali
◯ Malta
◯ Marshall Islands
◯ Martinique
◯ Mauritania
◯ Mauritius

◯ Mayotte
◯ Mexico
◯ Micronesia
◯ Moldova
◯ Monaco
◯ Mongolia
◯ Montenegro
◯ Montserrat
◯ Morocco
◯ Mozambique
◯ Myanmar
◯ Burma
◯ Namibia
◯ Nauru
◯ Nepal
◯ Netherlands
◯ Netherlands Antilles
◯ New Caledonia
◯ New Zealand
◯ Nicaragua
◯ Niger
◯ Nigeria
◯ Niue
◯ Norfolk Island
◯ Northern Mariana Islands
◯ Norway
◯ Oman
◯ Pakistan
◯ Palau
◯ Palestine
◯ Panama
◯ Papua New Guinea
◯ Paraguay
◯ Peru
◯ Philippines
◯ Pitcairn
◯ Poland
◯ Portugal
◯ Puerto Rico
◯ Qatar
◯ Réunion
◯ Romania
◯ Russia
◯ Rwanda
◯ Saint Helena, Ascension and Tristan da Cunha
◯ Saint Kitts and Nevis
◯ Saint Lucia
◯ Saint Pierre and Miquelon
◯ Saint Vincent and the Grenadines
◯ Samoa
◯ San Marino
◯ Sao Tome and Principe
◯ Saudi Arabia
◯ Senegal
◯ Serbia
◯ Seychelles
◯ Sierra Leone
◯ Singapore
◯ Slovakia
◯ Slovenia
◯ Solomon Islands
◯ Somalia
◯ South Africa
◯ South Georgia and the South Sandwich Islands
◯ Spain
◯ Sri Lanka
◯ Sudan
◯ Suriname
◯ Svalbard and Jan Mayen
◯ Swaziland
◯ Sweden

◯ Switzerland
◯ Syria
◯ Taiwan
◯ Tajikistan
◯ Tanzania
◯ Thailand
◯ Timor-Leste
◯ Togo
◯ Tokelau
◯ Tonga
◯ Trinidad and Tobago
◯ Tunisia
◯ Turkey
◯ Turkmenistan
◯ Turks and Caicos Islands
◯ Tuvalu
◯ Uganda
◯ Ukraine
◯ United Arab Emirates
◯ United Kingdom
◯ United States
◯ United States Minor Outlying Islands
◯ Uruguay
◯ Uzbekistan
◯ Vanuatu
◯ Venezuela
◯ Vietnam
◯ Virgin Islands, British
◯ Virgin Islands, U.S.
◯ Wallis and Futuna
◯ Western Sahara
◯ Yemen
◯ Zambia
◯ Zimbabwe

Please enter your country of origin.

◯ I'd rather not say
◯ Afghanistan
◯ Albania
◯ Algeria
◯ American Samoa
◯ Andorra
◯ Angola
◯ Anguilla
◯ Antarctica
◯ Antigua and Barbuda
◯ Argentina
◯ Armenia
◯ Aruba
◯ Australia
◯ Austria
◯ Azerbaijan
◯ Bahamas
◯ Bahrain
◯ Bangladesh
◯ Barbados
◯ Belarus
◯ Belgium
◯ Belize
◯ Benin
◯ Bermuda
◯ Bhutan
◯ Bolivia
◯ Bosnia and Herzegovina
◯ Botswana
◯ Bouvet Island
◯ Brazil
◯ British Indian Ocean Territory
◯ Brunei
◯ Bulgaria
◯ Burkina Faso
◯ Burundi
◯ Cambodia
◯ Cameroon
◯ Canada
◯ Cape Verde
◯ Cayman Islands
◯ Central African Republic
◯ Chad
◯ Chile
◯ China
◯ Christmas Island
◯ Cocos (Keeling) Islands
◯ Colombia
◯ Comoros
◯ Congo
◯ Cook Islands
◯ Costa Rica
◯ Côte d'Ivoire
◯ Ivory Coast
◯ Croatia
◯ Cuba
◯ Cyprus
◯ Czech Republic
◯ Denmark
◯ Djibouti
◯ Dominica
◯ Dominican Republic
◯ Ecuador
◯ Egypt
◯ El Salvador
◯ Equatorial Guinea
◯ Eritrea
◯ Estonia
◯ Ethiopia

◯ Falkland Islands (Malvinas)
◯ Faroe Islands
◯ Fiji
◯ Finland
◯ France
◯ French Guiana
◯ French Polynesia
◯ French Southern Territories
◯ Gabon
◯ Gambia
◯ Georgia
◯ Germany
◯ Ghana
◯ Gibraltar
◯ Greece
◯ Greenland
◯ Grenada
◯ Guadeloupe
◯ Guam
◯ Guatemala
◯ Guernsey
◯ Guinea
◯ Guinea-Bissau
◯ Guyana
◯ Haiti
◯ Heard Island and McDonald Islands
◯ Holy See (Vatican City State)
◯ Honduras
◯ Hong Kong
◯ Hungary
◯ Iceland
◯ India
◯ Indonesia
◯ Islamic Republic of
◯ Iraq
◯ Ireland
◯ Isle of Man
◯ Israel
◯ Italy
◯ Jamaica
◯ Japan
◯ Jersey
◯ Jordan
◯ Kazakhstan
◯ Kenya
◯ Kiribati
◯ Korea (North)
◯ Korea (South)
◯ Kuwait
◯ Kyrgyzstan
◯ Lao People's Democratic Republic
◯ Latvia
◯ Lebanon
◯ Lesotho
◯ Liberia
◯ Libya
◯ Liechtenstein
◯ Lithuania
◯ Luxembourg
◯ Macao
◯ Macedonia
◯ Madagascar
◯ Malawi
◯ Malaysia
◯ Maldives
◯ Mali
◯ Malta
◯ Marshall Islands
◯ Martinique
◯ Mauritania
◯ Mauritius

◯ Mayotte
◯ Mexico
◯ Micronesia
◯ Moldova
◯ Monaco
◯ Mongolia
◯ Montenegro
◯ Montserrat
◯ Morocco
◯ Mozambique
◯ Myanmar
◯ Burma
◯ Namibia
◯ Nauru
◯ Nepal
◯ Netherlands
◯ Netherlands Antilles
◯ New Caledonia
◯ New Zealand
◯ Nicaragua
◯ Niger
◯ Nigeria
◯ Niue
◯ Norfolk Island
◯ Northern Mariana Islands
◯ Norway
◯ Oman
◯ Pakistan
◯ Palau
◯ Palestine
◯ Panama
◯ Papua New Guinea
◯ Paraguay
◯ Peru
◯ Philippines
◯ Pitcairn
◯ Poland
◯ Portugal
◯ Puerto Rico
◯ Qatar
◯ Réunion
◯ Romania
◯ Russia
◯ Rwanda
◯ Saint Helena, Ascension and Tristan da Cunha
◯ Saint Kitts and Nevis
◯ Saint Lucia
◯ Saint Pierre and Miquelon
◯ Saint Vincent and the Grenadines
◯ Samoa
◯ San Marino
◯ Sao Tome and Principe
◯ Saudi Arabia
◯ Senegal
◯ Serbia
◯ Seychelles
◯ Sierra Leone
◯ Singapore
◯ Slovakia
◯ Slovenia
◯ Solomon Islands
◯ Somalia
◯ South Africa
◯ South Georgia and the South Sandwich Islands
◯ Spain
◯ Sri Lanka
◯ Sudan
◯ Suriname
◯ Svalbard and Jan Mayen
◯ Swaziland
◯ Sweden

	◯ Switzerland<br>◯ Syria<br>◯ Taiwan<br>◯ Tajikistan<br>◯ Tanzania<br>◯ Thailand<br>◯ Timor-Leste<br>◯ Togo<br>◯ Tokelau<br>◯ Tonga<br>◯ Trinidad and Tobago<br>◯ Tunisia<br>◯ Turkey<br>◯ Turkmenistan<br>◯ Turks and Caicos Islands<br>◯ Tuvalu<br>◯ Uganda<br>◯ Ukraine<br>◯ United Arab Emirates<br>◯ United Kingdom<br>◯ United States<br>◯ United States Minor Outlying Islands<br>◯ Uruguay<br>◯ Uzbekistan<br>◯ Vanuatu<br>◯ Venezuela<br>◯ Vietnam<br>◯ Virgin Islands, British<br>◯ Virgin Islands, U.S.<br>◯ Wallis and Futuna<br>◯ Western Sahara<br>◯ Yemen<br>◯ Zambia<br>◯ Zimbabwe
Please enter your occupational status.	◯ Full time<br>◯ Part time<br>◯ Unemployed<br>◯ Retired<br>◯ Student<br>◯ None of the above / I'd rather not say
Please enter your job title.	______________________________<br>(If you'd rather not say, just leave this field open.)
Please enter your annual net income.	______________________________<br>(If you'd rather not say, just leave this field open.)

What is the highest degree or level of school you have completed? If you are currently enrolled, please provide the highest degree received.

- ◯ No schooling completed
- ◯ Nursery school to 8th grade
- ◯ Some high school (no diploma)
- ◯ High school graduate (diploma or the equivalent)
- ◯ Some college credit (no degree)
- ◯ Trade/technical/vocational training
- ◯ Associate degree
- ◯ Bachelor's degree
- ◯ Master's degree
- ◯ Professional degree
- ◯ Doctorate degree
- ◯ None of the above / I'd rather not say

Please specify what type of diabetes applies to you.

- ◯ Type 1 diabetes
- ◯ Type 2 diabetes
- ◯ Gestational diabetes
- ◯ Other
- ◯ I don't know

Please enter your date of diagnosis. (D-M-Y)

____
(If you are unsure, please give an estimate or leave this field free.)

Please enter the date when DIY APS was started. (D-M-Y)

____
(If you are unsure, please give an estimate or leave this field free.)

Which type of DIY APS do you use regularly? Multiple options are possible.

- ☐ OpenAPS
- ☐ AndroidAPS
- ☐ Loop
- ☐ Other
- ☐ I don't know / I'd rather not say

If you are using an "other" system, please specify what system you are using.

____

If available, please give your last 3 HbA1c results from BEFORE DIY APS was used regularly.

Last HbA1c result from before DIY APS was started:

____
(Please use % as a dimension unit. If you are unsure, please leave this field free.)

Second-last HbA1c result from before DIY APS was started:

____
(Please use % as a dimension unit. If you are unsure, please leave this field free.)

Third-last HbA1c results from before DIY APS was started:

____
(Please use % as a dimension unit. If you are unsure, please leave this field free.)

If available, please give your Time in Range (percentage of sensor glucose within 70 - 180 mg/dl or 3,9 - 10,0 mmol/l) from BEFORE DIY APS was used regularly.

____
(Please use % as a dimension unit. If you are unsure, please leave this field free.)

If available, please give your last 3 HbA1c results from AFTER DIY APS was used regularly.

First HbA1c result AFTER the start of DIY APS:

____
(Please use % as a dimension unit. If you are unsure, please leave this field free.)

Second HbA1c result AFTER the start of DIY APS:

____
(Please use % as a dimension unit. If you are unsure, please leave this field free.)

Third HbA1c result AFTER the start of DIY APS.

____
(Please use % as a dimension unit. If you are unsure, please leave this field free.)

If available, please give your Time in Range (percentage of sensor glucose within 70 - 180 mg/dl or 3,9 - 10,0 mmol/l) from AFTER DIY APS was used regularly.

____
(Please use % as a dimension unit. If you are unsure, please leave this field free.)

Please tell us the approximate amount you have to pay each year for extra technology (such as server costs, hardware) to make DIY APS possible. Please convert your local currency to US-Dollar for comparision.

____

What motivated you to build a Do-it-yourself Artificial Pancreas system for yourself? Indicate your level of agreement with each statement by ticking one of the responses.

I built a DIY APS ...

	Fully applies	Largely applies	Partially applies	Does rather not apply	Does not apply at all
To achieve better overall glycaemic control.	○	○	○	○	○
To reduce the occurrence of acute complications (such as hypoglycaemia and hyperglycaemia).	○	○	○	○	○
To reduce the long-term complication risk (such as diabetic nephropathy, neuropathy, retinopathy etc.).	○	○	○	○	○
To put diabetes management more on auto-pilot and interact less frequently with the therapy system.	○	○	○	○	○
To improve my own sleep quality.	○	○	○	○	○
To improve sleep quality of partner/parents/family.	○	○	○	○	○
To increase my own life expectancy.	○	○	○	○	○
Due to the lack of availability of commercial closed loop systems in my country.	○	○	○	○	○
Due to the costs of commercially available closed loop systems (too expensive or not reimbursed by the healthcare system).	○	○	○	○	○
As commercially available closed loop systems are not suitable for my / my child's individual needs.	○	○	○	○	○
As therapy goals could not be achieved with the available therapy options.	○	○	○	○	○
Due to lack of medical support from the diabetes team (endocrinologist, diabetes educator, nurse, dietitian).	○	○	○	○	○

Due to lack of psychosocial support from health care professionals.	◯	◯	◯	◯	◯
Out of curiosity (technical or medical interest).	◯	◯	◯	◯	◯

Main other reason(s) (if any)

____
(max 500 words)

If you would like, please share your personal story about why you decided to build your own artificial pancreas system and how you got started. Feel free to share any experiences that had a significant impact on how you manage your diabetes as well. This story can be as short or as long as you wish.

When reflecting on your personal DIY closed loop story, you may want to consider the following:
When did you first hear about DIY closed loop systems and how did you look for further information?
Were there any key events or experiences that were a factor in your decision to begin closed looping?
Was there anyone else involved in helping you come to decision to begin DIY closed looping? For example a friend, family member or an online support group?
What were your emotions in the lead up to building your DIY closed loop system? For example, had you any major hopes or fears?

____
(max 1000 words)

Finally, describe some of the changes (if any) you experienced in your day-to-day life following successfully building the closed loop system. Did you experience any difficulties in making the transition to DIY closed looping?

____
(max 1000 words)

Thank you very much for your participation!

The OPEN research consortium.
